# Trust: an essential condition in the application of a caregiver support intervention in nursing practice

**DOI:** 10.1186/s12888-017-1209-2

**Published:** 2017-02-02

**Authors:** Marian I. Zegwaard, Marja J. Aartsen, Mieke HF Grypdonck, Pim Cuijpers

**Affiliations:** 1 Altrecht Mental Health Care, Gedachtengang 1, 3705 WH Zeist, The Netherlands; 2 NOVA Norwegain Social Research, Oslo and Akershus University College, Postbox 4, St. Olavs plass, Oslo, 0130 Norway; 3 University Centre for Nursing and Midwifery, Ghent University, De Pintelaan 185, block 5K3, 9000 Ghent, Belgium; 4 Department of Clinical, Neuro and Developmental Psychology, VU University Amsterdam, Van der Boechorststraat 1, Amsterdam, 1081, BT The Netherlands

**Keywords:** Older adults, Severe mental illness, Mental health care nurses, Nursing practice, Caregiver support

## Abstract

**Background:**

The recent policy of deinstitutionalization of health care in Western countries has resulted in a growing number of people - including elderly - with severe mental illness living in the community where they rely on families and others for support in daily living. Caregiving for partners, parents, children, and significant others can be a stressful experience and has been associated with psychosocial problems and poorer physical health. To support caregivers, a new, complex, nurse-led caregiver - centered intervention was developed. The intervention focuses on preventing deterioration in the wellbeing of caregivers. The objective of this study is to obtain a better understanding of the potentials of this new intervention.

**Methods:**

We applied an interpretative qualitative field study at two Dutch mental health care institutes. Thirteen caregivers participated in a one-time semi-structured interview.

**Results:**

From the caregivers’ perspective, a trusting relationship between caregivers and the mental health nurse is an essential condition for the depth and hence the effectiveness of the caregiver-centered counseling intervention. In this trusting relationship three overlapping and mutually reinforcing phases were identified (1) phase of engagement, (2) recognition of personal needs and (3) hope and optimism. Each phase encompasses key experiences that enhanced trust in that phase.

**Conclusions:**

Collaborative relationships between caregivers and mental health nurses provide a framework in which the mental health nurse can assess and help not only patients but also caregivers to gain insight into their situation and take on new roles and responsibilities in ways that promote their wellbeing.

**Electronic supplementary material:**

The online version of this article (doi:10.1186/s12888-017-1209-2) contains supplementary material, which is available to authorized users.

## Background

Older patients with severe mental illness often have to rely on family and friends (hereafter caregivers) for support and help in their daily activities. Severe mental illness such as schizophrenia, bipolar disorder, depression and anxiety disorders have a serious impact on the daily life of caregivers. These caregivers run an increased risk of developing mental disorders especially depression and anxiety [1–4]. These caregivers may also suffer from more stress-related somatic health problems, such as high blood pressure, cardio-vascular disease or diabetes [5–7]. To prevent severe deterioration of their quality of life - leading to a possible withdrawal from caregiving - these caregivers need support [8, 9].

Even though burden and increased psychosocial problems are seen in virtually all caregivers, the impact of the patient’s mental illness on the daily life of caregivers of older adults with severe mental illness deserves special attention. This is partly because these diseases often have little prospect of improvement and are more susceptible to relapse and/or complications due to physical frailty in both patient and caregiver. Moreover, the impact often has negative consequences for family functioning even across several generations [10–14] The consequences of caregiving are most readily seen in a deteriorating quality of the relationship with the patient and in the psychosocial well-being of the caregiver. In fact, all levels of interaction between caregivers, care-receivers and their social environment are affected by caregiving [14]. Relationships become unequal and brittle. Accordingly, the prevention of emotional distress, the enhancement of psychosocial wellbeing and the alleviation of entrapment among these caregivers is important [13–15].

Clinicians and researchers have devised many methods of trying to help caregivers. These include education and training programs, support groups, counselling, multicomponent interventions and E-health programs. Meta-analysis and systematic reviews on the effects of caregiver support interventions are mostly conducted among inpatients with dementia and schizophrenia and the outcome measures are improvement of patient’s wellbeing, caregiver burden and wellbeing. These studies fail to address crucial factors that may help to improve caregiver wellbeing. Psycho-education, the most commonly used intervention strategy in caregiver intervention research, shows a small and heterogeneous body of evidence with an emphasis on improved knowledge of the illness, knowledge of useful coping skills and overall social functioning [16–18]. Multicomponent interventions, i.e. interventions that combine different strategies to provide caregivers with various services and supports, tend to generate larger effects than narrowly focused interventions [19]. Still the outcomes vary from significant effects on caregiver burden, well-being, and increased knowledge to no effects on depression or patient symptoms [17, 20] and limited effects on the risk of hospitalisation [21].

Reviews of psychosocial interventions [17, 22, 23] conclude that elements for success are practical support for the caregiver, involvement of the extended family, structured individual counselling, and flexible provision of a consistent professional to provide long-term support. A study of the effect of psychotherapy on improved wellbeing among clinically depressed elderly caregivers demonstrates that these elderly caregivers, who in fact can be regarded as patients, benefit from time limited psychotherapy [17, 24].

Already existing interventions are often too briefly elaborated and pay little attention to decreased wellbeing in caregivers. Based on a method developed by Van Meijel and colleagues (2004) [25], a complex, nurse-led caregiver support intervention for caregiver-centered counseling was developed for mental health nurses (MHN) to support caregivers in face-to-face conversations. This intervention includes a focus on the consequences of the patient’s mental illness for the caregiver’s personal life, and management of difficult behavior and psycho-education within the context of “the consequences for the personal lives” of the caregivers. The intervention is tuned to the needs of caregivers who often perceive no other choice than to continue to provide care. The intervention can be used at any time during the patient’s treatment. Accordingly, the objective of the intervention is to contribute to the prevention of a decline in wellbeing due to psychosocial problems caused by the existential impact of the consequences of the mental illness on the everyday life of the caregiver [14].

In the present article, we describe the lived experience of caregivers who received this caregiver centered intervention, for the first time. We opted for a qualitative explorative field study in order to gain insight into caregivers’ experiences of the intervention and the underlying processes.

## Methods

### Description of the intervention protocol

The intervention, a caregiver-centered intervention protocol, is intended to support those caregivers of older adult patients with a severe mental illness who feel a moral or factual obligation to continue giving care to older adult patients with severe mental illness despite the burden involved. This burden may involve existential issues that affect their own lives and their relationship with the patient. The caregiver–centered intervention is shaped by knowledge of the caregiving context and acknowledgement of the caregiver’s problems and needs. The intervention protocol instructs the mental health nurse (MHN) how to arrive at a joint basis for the individual support of mainly caregivers who “perceive no freedom of choice to quit caregiving” [14]. During counseling sessions, the mental health nurse aims at optimizing interpersonal functioning and improving the psychosocial wellbeing of those caregivers. As a result, the caregivers should feel more competent about their ability to adapt to the consequences of the mental illness in their personal lives, and act accordingly. These counseling sessions have the nature of an open discussion about frequently occurring problem areas. To address conflicts associated with dual roles for MHN - as the MHN supports both the caregiver and the patient - the MHN explains the content of the caregiver-centered intervention to both the patient and the caregiver, and mutual expectations are discussed. Regular alignment between the goals of the patient and the expectations of the caregiver is part of the protocol.

The intervention protocol consists of three phases;

- The preparatory phase, in which caregiver and patient are given information about the patient’s illness and treatment and about the possible impact on the caregiver’s life. An appointment is made to assess caregiver needs.
- The second phase, in which the mental health nurse coaches the caregiver on issues that appear to be frequently occurring problem areas for the caregiver’s wellbeing: tension in the relationship; role transitions; grief; loneliness and isolation, and the feeling of entrapment.
- The third phase has an emphasis on further energizing the caregiver’s own competence and validating what the caregiver has achieved. Also, the need for follow-up conversations is addressed.

The intervention protocol offers the mental health care nurses a clear structure for the execution of the intervention but also provides sufficient space for adapting the method to the individual characteristics and needs of the caregiver and the specific context in which the support is provided. To learn how to use the intervention in daily practice the MHNs follow training to learn the content of the intervention, the theoretical background of the content and the application of the intervention in daily nursing practice. During peer meetings the MHNs discuss their new role, the way they operate with respect to both the caregiver and the patient, and potential dilemmas they can encounter as a consequence of this.

### Design

In a pilot study the lived experience of caregivers when receiving the new intervention in daily nursing practice is explored. Therefore an exploratory, interpretative qualitative field study was chosen [26]. Using this method meant experiences could be described, and at the same time we could uncover the processes involved.

### Procedure and data collection

#### Process of approaching potential participants

Purposive sampling was used. First, the researcher explained the aim and design of the study to a group of mental health nurses from two large mental health care organizations in the Netherlands which focus on the treatment of adults with severe mental illness. Second, MHNs who were interested in caregiver support were asked to participate in this pilot study. Nine MHNs invited two caregivers of two different patients, who, despite the lengthy duration and complexity of care, had persevered with caregiving. The caregivers were eligible for inclusion if they spoke Dutch, were the primary caregiver (as judged by the mental health nurse) of an older community dwelling or temporarily hospitalized patient with a severe mental illness; the patient was at least 60 years of age and the caregiver had been caring for the patient for at least 6 months. Excluded were caregivers of older adults with manifest cognitive impairment, including dementia. The MHNs explained the content of the study to these caregivers informed them about the qualitative interview at the end of the study period and invited them for participation in the caregiver centered intervention.

*Participants* A total of 18 caregivers accepted the invitation from the MHN to experience the intervention and were willing to participate in an interview about their experiences during coaching. The participating caregivers knew that their MHN was learning a new method and that they had to practice in real patient–caregiver situations. By the end of the pilot period, 13 of the 17 participating caregivers (one caregiver died) participated in a semi-structured interview conducted by the first author. Two caregivers did not participate due to relapse of the patient and one caregiver was (according to the mental health care nurse) too heavily burdened to participate. The interviews lasted between 60 and 90 min and were held in the patient’s absence and at a location of the caregiver’s choosing. The researcher was not known to the caregiver before the beginning of the study.

Characteristics of the overall sample: the 13 participating caregivers were diverse in terms of gender, years of caregiving and caregiver category (type 1 or type 2a or type 2b). Type 1 or type 2a or 2b refers to the concept “freedom of choice to quit caregiving” with type 1 caregiver generally experiencing gain, whereas both types 2 generally experience loss, which puts the latter groups typically at risk of becoming overloaded [14]. These characteristics may have influenced their perspectives and the content of their needs. The caregivers’ characteristics are presented in table 1.

**Table 1:** Characteristics of the interviewed caregivers

Characteristics		*N* = 13 Agreed to participation
Gender	Male	6
	Female	7
Caregiver’s Age	55-60	1
	61-65	2
	66-70	5
	71-75	4
	76-80	1
Patient’s Age	61-65	2
	66-70	4
	71-75	5
	76-80	2
Type of relationship	Married	11
	Sisters	2
Type of caregiver	Type 1	1
	Type 2a	6
	Type 2b	5
Duration of care	12 - 24 months	4
	02 - 10 years	5
	20 - 45 years	4

### Data collection

The interviews of 12 caregivers were audiotaped. One respondent was willing to participate but refused to have the interview recorded. In this case, notes were made on paper. During the interviews the caregivers were encouraged to tell their story. All interviews started with an open question: What did the conversations with the MHN mean to you? A topic guide was used with questions about the actual content of their conversations with the MHN and about the desirability of the content of the conversations (additional file 1). The latter questions also focused on the structure and process of the conversations and the transferability of the content to their daily life with the person they cared for.

### Data analysis

The analysis of the individual interviews was conducted in a cyclical process in which two complementary and intertwined strategies were used, namely coding and thinking theoretically [27, 28, 29]. Thinking theoretically means formulating interpretative concepts and identifying relations between these concepts. This involves bringing together knowledge we had from our experience, previous research and the study of the literature. In doing so, the analysis went beyond the individual case. A research team of three members (MIZ, MA, MG), including the interviewer (MIZ) was involved in process of data analysis. MIZ and MG were involved in the entire process of data analysis while MA challenged any potential self-deceptions of MIZ and MG during critical dialogue. The interviewer (MIZ) and a member of the research team (MG) analyzed the interviews. Peer review was conducted in order to enhance validity. The interviewer (MIZ) marked text fragments representing a reaction of the caregivers to the conversations with the MHN. Then the interviewer (MIZ) and the one member of the research team (MG) discussed the marked text fragments to achieve consensus regarding the interpretation of the text fragments by considering several possible meanings. This guided constant comparative analysis. MIZ checked all the insights and interpretations against existing data and new material. Before coding the text fragments, MIZ and MG consulted MA to discuss the interpretation of the data so far. In the interpretation of the data possible bias was taken into account. Here explicit attention was given to possible negative experiences of caregivers. Subsequently, all these text fragments were coded and a code tree was developed. Analytical thoughts and ideas with respect to the data were discussed in order to reach an understanding of the respondent’s point of view [30]. The two researchers (MIZ, MG) worked towards consensus in interpretation, comparing text fragments within and between cases. MIZ checked the insights that emerged from the discussions against the data by referring back to the transcripts, to interpretations at an earlier stage of the analysis and to field notes. The transparency of the analytical process and verifiability of the research were enhanced by using field notes and putting forward provisional interpretations supported by the relevant text fragments. During ongoing discussions these text parts were recoded and the code tree was redesigned; subsequently, common themes for structuring concepts were identified. Then the third researcher (MA) was consulted again. She read a selection of the interviews and checked the final interpretation and conclusions against the data. Again, explicit attention was given to possible negative experiences in all phases of the trusting relationship. This iterative process constituted researcher triangulation and increased both the depth and reliability of the analysis. The software program MAXQDA was used to increase insight into content, meaning and recurrence of themes. This study revealed a central organizing construct, i.e. a trusting relationship based on three partly overlapping and mutually reinforcing phases along with key experiences that enhanced trust in each phase. Within our sample saturation was reached with respect to these phases and key experiences. In order to adhere as closely as possible to the meaning of the quotes three people were involved in the translation of the quotes from Dutch to English. First the interviewer (MIZ) translated the quotes followed by successively a native speaker and one of the researchers (MG) who checked for accuracy.

## Results

### Trusting relationship

The analysis revealed that from the perspective of the caregivers, a trusting relationship between caregivers and the MHN is an essential condition for the depth and hence the effectiveness of the caregiver-centered intervention. In this trusting relationship, which evolved during a process of bipartite exchange between the caregiver and the MHN, three partly overlapping and mutually reinforcing phases were identified (1) engagement, (2) recognition of personal needs and (3) hope and optimism (table 2). Each phase encompasses key experiences that enhanced trust in that phase. These phases were not linear but mutually reinforcing sub-processes that could overlap, and in which trust was both the outcome and basis of (further) collaboration. Hence trust intensified in strength and depth in each phase, with the trusting relationship spiralling upwards and giving rise to hope and optimism instead of to feelings of fatalism. Apparently, several supportive elements underpin this evolvement of the relationship: namely the caregivers believe that the MHN wants what is best for both caregiver and patient; the caregivers assume that their vulnerability is respected, their trust is not violated, and that promises are kept.

**Table 2:** Phases and key experiences of a trusting relationship

Phases	Key experiences
Phase of Engagement	Being understood
	Someone you can depend on
Phase of Recognition of personal needs	Awareness of feelings and thoughts
	Awareness of change
Phase of Hope and Optimism	Regaining control
	Regaining oneself as a significant person

“(79). I talked openly to the nurse and we are always honest with one another. I discuss everything with him and we can be truthful with one another. When you can look each other straight in the eye, something clicks between you. Then the patient also benefits”.

None of the caregivers criticized the intervention. The phases and key experiences are discussed below.

### Phase of engagement

Trust was awakened when the caregivers realized the MHN deliberately wanted to spend time with them, listening to their pain and trying to anticipate their needs. This trust was initiated when the MHN, as a first step in the intervention, explicitly invited the caregiver for a conversation (without the patient) about the impact of caregiving on his/her personal life. With this invitation, the caregiver realized that the MHN was there not only to support the patient, but also for the caregiver. Thanks to this “invitation”, caregivers no longer felt they were merely the anonymous person behind the patient.

#### Being understood

The feeling that the MHN is “willing to understand” what is happening in their daily lives created a foundation to building a trusting relationship and establishing a collaborative process that caregivers found supportive and helpful in regaining control over their own lives. At first the caregiver felt surprised that a person (with a professional background) took a genuine interest in the day-to-day issues that concerned them. Caregivers felt they were given recognition by the pro-active attitude of the MHN who initiated several appointments even when no actual problems relating to caregiving needed to be solved. This exploration of the caregiver’s subjective experience is perceived as a validation of themselves as a person. The caregiver experienced true interest from the MHN when the MHN helped to address problems before they escalated. The use of practical advice that suited the caregiver’s situation increased the caregiver’s feeling of trust because it indicated the MHN’s understanding of what the caregiver was going through.

“(82) R: I thought it was very nice that I was the one who it was all about. All those months, even a whole year long, he (husband) was the center of everything. I did not envy him that but I had the feeling of “hello I’m here too”.

Mutual trust was enhanced by this telling of one’s story in an open conversation with the MHN who respects the caregivers’ opinions and motives for caring. Caregivers realized that thanks to their professional expertise, the MHNs understood their hopelessness as the result of the impact of the illness and did not misinterpret it as being due to “evil thoughts”.

#### Someone you can depend on (an ally)

Careful listening, together with a non-judgmental supportive attitude, made the caregiver feel more comfortable in disclosing sensitive information to the MHN. As a result, the caregivers felt they had an ally. By allowing the caregivers to tell their story, and by looking behind their initial verbal presentation of the content of their care for the patient, the MHN became someone the caregivers could rely on and who could be trusted. This trust was the foundation for a trusting relationship in which caregivers started sharing intimate thoughts, uncertainties, difficulties, hopes and dreams for the future. This sharing with the MHN, who monitored both their wellbeing and that of the patient, was not perceived as betrayal of the patient but provided relief to the caregivers. Another sign of engagement and interest which enhanced the sense of having an ally was the fact that the MHNs also took responsibility for assessing and attending to the needs of the patient. This proved to further bolster the sense of alliance and further optimized the relationship.

“(008) R: The nice thing about talking to the mental health nurse is that I can speak freely and she is there to help me too”.

The content of the conversations gave the caregivers confidence that the support was given skillfully and was adequate for their needs. Our data also showed that some caregivers who participated would have preferred more in-depth support. In these cases the MHN did recognize the problems but did not explore them as the caregiver would have liked. In the cases in which the MHNs reflected on the caregiver’s story and did explore their personal needs, the caregiver expressed a sense of feeling safe and comfortable, and this appeared to be a good basis for progression of trust to the next phase.

### Phase of recognition of personal needs

The MHN’s validation of the caregiver’s story allowed the caregivers to be open and to explore and reflect on their story, working towards the recognition of their personal needs and considering possible changes. By doing so, caregivers first learned to reflect on their feelings and thoughts, learning to see themselves as a person as well as a caregiver and then becoming aware of possible (opportunities for) change.

### Awareness of feelings and thoughts

The participating caregivers were too involved in their care for the patient to take proper care of themselves. Careful, non-judgemental listening by the MHN allowed the caregivers to look at themselves more objectively, without having to defend their actions. Caregivers experienced the conversations as a safe foundation for collaborative discussions which was understood as an opportunity to make sense of their own experiences and to learn how these feelings were related to caregiving.

“(76) R: After talking to the MHN I began to feel calmer. Thanks to these quite deep conversations we (caregiver - MHN) got to know each other better. The conversations were beneficial for me. I mean free speech, I need that sometimes and I dared to raise all sorts of things (emotions) that bothered me. Did you ever talk about your feelings with someone before? R: No”.

“(78) R: We talked about grief. About my difficult life with my wife who has troublesome moods. Yes that is at times very difficult and very sad. Yes we talked about it “.

This made them realize that they had been ignoring their own needs. Caregivers became aware of the fact that they could hardly remember what their personal interests and hobbies were before they had become a caregiver. It appeared that the caregivers often had no clue as to how to rebalance their personal lives against their responsibilities for the patient.

“(78) I: As a result of the conversations with the MHN do you act differently? R: Yes. Yes, I leave things now. I’m getting older and I should accept that. And I have someone who helps with the housekeeping. I think that is nice. All this happened because of the conversations with the MHN. I see things differently now and I’m going to do things differently. For instance, I clean the windows every three months instead of every two months. Things like that, I think yes, they are no longer the most important things in life. I: What do you think is important? R: Me - myself. Now I do puzzles during the day. Before, I would really not have done that. Or I might read a little. Just relax “.

#### Awareness of change

In their growing awareness of themselves as individuals, the caregivers experienced the MHN’s support as a reliable guide in the search for new ways to deal with the impact of the patient’s mental illness on their life. Trust facilitated collaborative in-depth discussions about risk taking (meaning new behavior). The MHN provided the caregivers with reassurance by normalizing their feelings, by not criticizing their thoughts or behavior and by legitimizing experimentation with new behavior towards themselves and the patient.

As the caregivers felt they could now rely on the MHNs’ support, they looked to the future with more confidence and with less anxiety. This sense of relief was further optimized when the caregivers got to experiment with new behavior, as fostered and coached by the MHN. Caregivers experienced a sense of shared responsibility while experimenting with new behavior. By legitimizing this kind of experimentation, the trusting relationship was further optimized. One of the caregivers responded thus:

“(76) I: Were you aware that you had taken on so much? R: I did realize it but I thought it was normal. I am a caring husband and many times I thought why shouldn’t I help her by taking things out of her hands? But I found it tough and now I say to myself: I should not do that. I must say it is very difficult to do otherwise. In the conversations I learned the importance of giving back responsibilities - both for myself and for my wife “.

Although things were placed in perspective and the relationship of mutual trust was further strengthened, thus providing safety and tranquillity, it was still difficult to use this new “broadened perspective” without the help of the MHN.

### Phase of hope and optimism

The helpful conversations about the caregivers’ experience and about new ways to handle their situation occurred in the context of a collaborative, trusting relationship which facilitated regain of control and of oneself as a significant person.

#### Regaining control

As confidence in the alliance with the MHN increased, the conversations were experienced as “relief time", or "time for themselves” in which the caregiver identified areas to work on, experienced learning, assumed control and accepted more responsibility in caring for themselves. Although most caregivers still experienced their life as burdensome, they appreciated the support of the MHN as a “complete experience” in which the collaborative discussions supported a growing awareness of a changed perspective where the future is concerned. Caregivers talked about hope that in the near future things might be a little different.

“(79) R: I talked separately with the MHN and we discussed all sorts of things. Before then I had no one and *I* had to figure things out all by myself. Trying to find out what is the matter and how I could help her? So I had a lot of questions that were not necessary, but I was a layman. Now I know I’m doing the right thing. I had to change my whole life because I am used to getting up at seven o’clock in the morning, and arriving home at seven in the evening. Now I was also doing all of the housework - cooking, cleaning, everything. I have two jobs actually. Recently I resumed one of my favorite hobbies. I started playing badminton again and I’m really enjoying it “.

#### Regaining a sense of oneself as a significant person

Caregivers talked about increased insight and confidence in their own ability to handle the impact of caregiving on their life. The trust relationship lessened feelings of being solely responsible and hence gave them hope that the future could be different. It helped them not to bottle up their emotions but to become stronger. Caregivers talked about personal growth, increased knowledge and the courage gained to try out new behavior. The insight into the causes of their mixed feelings was reassuring because they now could explain these feelings as a part of their caregiving role instead of in terms of who they had become. Although not all attempts at using new behavior were equally successful, the perspective of “possible change” gave them confidence that their lives could be about more than being (an absorbed) caregiver. The acknowledgement that the use of new behavior did not hurt the patient confirmed their hope for change.

“(82) R: I told him I’m your wife; I’m not your nurse. I wanted to separate the two roles. We had somebody in just for housework. He (husband) did not like strangers in our house. I also had to get used to that but as the MHN also says, you set your limits and act accordingly. It is difficult but necessary for me”.

The insights and the experiments with new behavior strengthened the caregivers’ awareness of themselves as a significant person. With regained self-respect and the acknowledgement of themselves as individuals, caregivers felt that they were able to influence the course of events.

## Discussion

In this study we found that, from the perspective of the caregivers, the trusting relationship between caregivers and the MHN is an essential condition for the caregiver-centered counseling intervention to become beneficial. Through our analysis we confirmed that this trusting relationship contributes to a better understanding of why the intervention is appreciated and accepted by the participating caregivers. Building a trusting relationship can also be considered as a process, that facilitates the depth and hence the effectiveness of the caregiver-centered counseling intervention. Irrespective of people’s gender, all caregivers appreciated the conversations and they eagerly used the opportunity to speak freely, as the conversations were held in the patient’s absence. They now felt the freedom to talk openly about their emotions arising from caregiving. This afforded them the opportunity to reflect on how to fulfill their caregiver role while making sense of their own life experiences. Originally the interviews were meant to explore the feasibility of the intervention, but the study gave good insight into the importance of a trusting relationship between the caregiver and the MHN. Research among patients and (mental health) nurses underpins our finding that collaborative a relationships between caregivers and MHNs provides a framework in which the MHN can assess and help not only patients but also caregivers in taking on new roles and responsibilities. This increases the likelihood that new activities and opportunities will be planned and carried out in ways that promote wellbeing rather than endanger empowerment [31–33].

The added value of building trusting relationships between caregivers and (mental health) nurses has so far received little attention in caregiver research. That the success of an intervention is reliant on the referring clinician’s (MHN’s) relationship with the caregiver is hardly discussed in intervention research [34]. As has been described in research with mentally ill patients, we assume that a trusting relationship is also important for the effectiveness of caregiver interventions. Possibly, similar steps in building a trusting relationship that were reported in earlier research [31, 32] might explain why individual support is more effective for caregiver burden and wellbeing than group support [17]. Research on the application of a relatively new form of caregiver intervention - EHealth - discusses the importance of a coach - which equates with a possible trusting relationship - for the effectiveness of the intervention and for combating the high drop-out rate [20]. Accordingly, discussing the merits of a trusting relationship as a factor in the success of caregiver interventions is advised.

The elements of successful caregiver support (structured individual counselling and flexible provision of a consistent professional), are in keeping with the essential role of mental health nurses in the care for both patients and their caregivers. The nurses’ intensive contact with patients and the likelihood of home visits contribute to their approachability for the caregiver and create opportunity for dialogue [35]. Mental health nurses witness the consequences of severe mental illness on the lives of both patient and caregiver from an insider’s perspective. This and the fact that mental illnesses are often severe and persistent mean that mental health nurses are likely to have many opportunities to attend to the personal needs of caregivers. These natural moments afford an important opportunity to ask the caregivers about their wellbeing and to assist them in supporting their relative’s treatment goals. Such structured individual support requires this inside knowledge about the caregiver's situation and insight into the factors and processes that determine the impact of caregiving on the caregiver’s daily life.

Although some of the participating MHNs reported that they were sometimes modest in applying the nursing protocol, the interviews demonstrated that the caregivers experienced their contact with the mental health nurse as an experience in which they felt heard and seen. The interviews afforted insight into the appreciation and acceptability of the intervention and we arrived at the conclusion that the participants felt that the content of the conversations was about topics that they recognized as a part of their life. Although in some of the interviews it was less clear whether the reported change is due to the feeling of support from the MHN, as some caregivers also attend a psychologist, some of the caregivers would have liked to deepen the conversations. They endorsed the topics that were discussed during the conversations. Considering the fact that not all topics are of equal importance to the caregivers it was not possible for them to comment on all topics of the intervention. On the basis of our findings, we argue that caregivers of older people with severe mental illness need the same elements of recognition and connectedness for strengthening their sense of self (identity), empowerment and overall life expectations (hope and optimism) as do patients [33, 36, 37]. Therefore a relational perspective as already described by Peplau (1989) [38] should not only be applied in patient-MHN relationships. These results from the pilot study resemble elements of person-centered therapy that echo the humanistic school of psychotherapy which formally began with Carl Rogers (1959) [39]. An unconditional positive attitude by MHN, empathic understanding of the internal frame of reference as well as a trusting relationship is in keeping with this caregiver -centered approach. Although the MHN is not a therapist, we argue that these elements contribute to the therapeutic efficacy of the nursing intervention.

### Limitations

We realize that this purposeful sample included only those caregivers who persevered with caregiving. These, mostly type 2 caregivers, represent a subgroup of caregivers who deal with the complex consequences of the mental illness in their daily life.

The selection of caregivers by the professionals involved may be considered to be a limitation of the pilot study, as all participating caregivers were approached by their mental health care nurse. It is possible that these professionals may have refrained from inviting certain caregivers if they thought that participation in the study could be too burdensome. Perhaps this could explain why the caregivers, despite the sometimes modest application of the intervention protocol, mentioned almost no negative experience with the intervention.

The prolonged involvement of the researcher, who is also a mental health nurse, comes with certain risks as the researcher felt a sense of responsibility for and sometimes identified herself with the desired outcome of the study. At the same time, the use of field notes, gathered during data collection, the use of memos and critical reflection sought and found in the research team, with the (participating) mental health nurses, reduced the risk of bias.

### Strengths

This study is qualitative in nature, hence causal relationships cannot be established [40]. To reduce limitations in strength and improve validity and generalisability, we conducted the analysis from the data through to the theory generation with a team of researchers from various disciplinary backgrounds. Transparency of the analytical process and verifiability of the research were enhanced by discussing provisional interpretations. The members of the research team critically questioned the assumptions of the researcher at several moments during data analysis. Reflection on the way the researcher might have led the interviews was also the subject of discussion. A consistent and thorough use of triangulation has limited the subjectivity of the interpretation of the data by challenging possible self-deceptions. Confirmability was enhanced by means of triangulation and verification of results by an independent researcher. The use of constant comparative analysis and the intertwined use of coding and theoretical thinking, familiarity with the culture of the participating organizations and the above- mentioned purposive sampling of the respondents and researcher triangulation all contribute to the credibility of the research.

## Conclusion

### Implications for nursing research

This study gives insight into the experience of caregiver support from the perspective of the caregiver. It is a pilot study that has shed light on the importance of the trusting relationship for the depth and outcome of the counseling sessions. More research is urgently needed to clarify whether the trusting relationship is a factor that is predictive in the success of caregiver interventions. The influence of gender differences on the experience of the caregiver-mental health nurse relationship could be an important issue for further research. In view of the emotions in the narratives and considering the tasks which they face, it appears that the male participants might need to step out of their traditional roles. This may be an interesting issue for further research. In this pilot study we did not ask the patients about the benefits of support to the caregiver for the patient’s wellbeing. To learn about the influence of the caregiver’s wellbeing on patient outcome is another subject for research. Another issue is that of validation of the caregiver’s story. In the interviews with the researcher the particpants again felt the need to tell their story and ask for reassurance and confirmation of their identity. Finally, an effect study with the piloted intervention is recommended.

### The intervention protocol in nursing practice

This pilot study makes clear that a trusting relationship with MHNs is a key variable in the support of caregivers. If a caregiver assessment suggests that a caregiver needs help, whether this is to enhance caregiving or to reduce psychological distress and improve wellbeing, caregiver-centered interventions should be considered as part of integrated services for people with severe mental health problems. The intervention runs parallel with the treatment of the patient and therefore can be applied to problems and specific phases of the illness and its impact for both caregiver and patient and their interpersonal relationship. The complexitty of caregiver dynamics means that counseling of the caregiver should be carried out by MHNs and professionals with skills in system approaches and family therapy. Based on these study results we will integrate these findings into the training of the intervention protocol. As the intervention protocol is applied to the caregivers of the MHNs’ patients, the MHN will sometimes experience a certain conflict of interest in supporting both patient and caregiver. In those cases the MHN needs to realize the impact of the illness on the caregiver’s psychological and physical health, and ask a colleague to have the conversations that are necessary for the caregiver’s wellbeing.

## Additional file

Additional file 1: Topic guide for interview with caregiver. A topic guide was used with questions about the actual content of the conversations between the caregivers and the MHN and about the desirability of the content of the conversations (additional file 1). (DOCX 17 kb)
