# Supplementary material for: Trust: an essential condition in the application of a caregiver support intervention in nursing practice
Source: BMC Psychiatry. 2017 Feb 2;17:47. doi: 10.1186/s12888-017-1209-2 (PMC5288942; doi:10.1186/s12888-017-1209-2)
Supplement: Additional file 1: — Topic guide for interview with caregiver. A topic guide was used with questions about the actual content of the conversations between the caregivers and the MHN and about the desirability of the content of the conversations (additional file 1). (DOCX 17 kb) [file 12888_2017_1209_MOESM1_ESM.docx]

Additional file 1. Topic guide for interview with caregiver

*Introduction:*

In recent months you have had several conversations with (name of) your mental health care nurse (MHN). In these conversations you talked about the impact of caregiving on your life and your personal wellbeing. Both of you talked about a number of topics. In this conversation now I would like to talk you through the meaning of these conversations for your personal situation. In other words, has it helped?

*General questions*

- How are you?
- Did you know what to expect from participating in the conversations?
- What is your overall feeling regarding the conversations?
- Did you get enough information about what to expect from the conversations?
- Were you able to tell your story?
- Can you tell me what topics you talked about?
- Did the content of the conversations fit your situation?
- Did participating in the conversations with the MHN help you?
- In what way did it help you? (Or what might be the reason it did not help you?)
- Have you gained better insight into the impact of caregiving on your life?
- What is the best thing you remember about the conversations?
- Have you ever received any advice? If so, what was this advice about?
- Why do you think caregivers evaluate the conversations positively?
- Are there any tips you can give to other caregivers who plan to participate in coaching?

*Desirability of the intervention*

- What would you like to keep in these conversations?
- Are there things you would like to change in the approach? If yes, why?
- Did you find this approach worthwhile for caregivers you know?
- Why or why not?
- How did you co-decide on the selection of topics to discuss?
- What support would you like to see continued?
- What topics would you like to have discussed more?
- Were there topics you never thought of talking about that turned out to be worthwhile? If so, which ones?
- Looking back, what subjects were less important to talk about?
- Looking back, what would you have wanted to have talked about but did not?
- What do you think is important in a training course for MHNs?
- What kind of support would you like?

*Evaluation of the structure and process of the conversations*

- What is your opinion about the number of conversations with (name of) MHN?
- What is your opinion about the duration of conversations with (name of) MHN?
- Were there topics you agreed to talk about but that were never discussed?
- What number of conversations do you find appropriate; more or less than took place?

*Applicability of the content of the conversations?*

- Were there any situations that made it difficult to fulfil your own needs?
- Did you find the information received useful?
  - in the short term?
  - in the long term?
- Do you feel that you have gained more control in your life and/ or your life with (name

.
